# Comparative efficacy of varicocelectomy and intrauterine insemination in varicocoele patients with mild semen abnormalities: An observational study

**DOI:** 10.1111/andr.70070

**Published:** 2025-05-30

**Authors:** Yanlin Ma, Weiming Deng, Tingting Wu, Qi Li, Yuanjie Li, Wenjie Mai, Lizheng Yan, Changwei Liang, Lingxiao Zhang

**Affiliations:** ^1^ Department of Reproductive Medicine The First Affiliated Hospital of Hainan Medical University Haikou China; ^2^ Hainan Provincial Key Laboratory for Human Reproductive Medicine and Genetic Research & Key Laboratory of Reproductive Health Diseases Research and Translation, Ministry of Education & Hainan Provincial Clinical Research Center for Thalassemia The First Affiliated Hospital of Hainan Medical University, Hainan Medical University Haikou Hainan China; ^3^ Department of Urology The First Affiliated Hospital of University of South China Hengyang Hunan Province China; ^4^ Hainan Modern Women and Children's Hospital Reproductive Medicine Haikou Hainan China; ^5^ Department of Urology Honghuagang District People's Hospital Zunyi City Guizhou Province China; ^6^ Department of Urology Sanya People's Hospital Sanya City Hainan Province China; ^7^ Department of Urology Hainan Western Central Hospital Danzhou City Hainan Province China

**Keywords:** intrauterine insemination (IUI), mild semen abnormalities, pregnancy outcomes, varicocoele, varicocelectomy

## Abstract

**Background:**

Varicocoele is a common cause of male infertility, affecting spermatogenesis through increased testicular temperature, venous stasis, and oxidative stress. Microsurgical subinguinal varicocelectomy improves semen quality, whereas intrauterine insemination is widely used for mild male factor infertility. The comparative efficacy of these treatments in varicocoele patients with mild semen abnormalities remains unclear.

**Objectives:**

To evaluate the efficacy of microsurgical subinguinal varicocelectomy and intrauterine insemination in improving clinical pregnancy and live birth rates in varicocoele patients with mild semen abnormalities and assess post‐operative improvements in semen parameters following microsurgical subinguinal varicocelectomy.

**Materials and methods:**

A retrospective cohort study involving 650 microsurgical subinguinal varicocelectomy patients from five medical centers and 700 intrauterine insemination patients from one center was conducted. Inclusion criteria included varicocoele diagnosed via ultrasonography, mild semen abnormalities (total motile sperm count ≥5 million), and at least one abnormal semen parameter. Primary outcomes were clinical pregnancy and live birth rates. Secondary outcomes included sperm concentration, motility, and total motile sperm count changes post‐microsurgical subinguinal varicocelectomy. Statistical analyses included chi‐square tests and logistic regression.

**Results:**

Microsurgical subinguinal varicocelectomy patients demonstrated significant improvements in sperm concentration (35.2–43.3 × 10⁶/mL), motility (26%–38%), and total motile sperm count (18.8–34.9 × 10⁶, *p* < 0.001). Clinical pregnancy and live birth rates were higher in the microsurgical subinguinal varicocelectomy group (35.23% and 31.08%) compared to the intrauterine insemination group (29.57% and 24.00%, *p* < 0.05). Multivariate analysis revealed that microsurgical subinguinal varicocelectomy significantly increased pregnancy (OR = 1.43, 95% CI: 1.12–1.83, *p* < 0.05) and live birth rates (OR = 1.56, 95% CI: 1.21–2.02, *p* < 0.05).

**Discussion and conclusion:**

Microsurgical subinguinal varicocelectomy significantly enhances semen quality and achieves superior clinical pregnancy and live birth rates compared to intrauterine insemination for varicocoele patients with mild semen abnormalities. These findings suggest that microsurgical subinguinal varicocelectomy is a more effective treatment option, highlighting the importance of individualized treatment strategies and supporting the preferential use of surgical intervention in this specific patient population.

## 1 INTRODUCTION

Globally, 15% of couples of reproductive age face fertility issues, with male factors accounting for approximately 50% of cases.^1^ Varicocoele is one of the most common causes of male infertility, with a prevalence of 10%–15% in the general male population, 35% in primary male infertility cases, 69%–81% in secondary infertility cases, and 25.4% among men with abnormal semen parameters.^2^, ^3^, ^4^ The adverse effects of varicocoele on male reproductive function are primarily attributed to increased testicular temperature, venous stasis, and oxidative stress, which collectively impair spermatogenesis.^5^

Microsurgical subinguinal varicocelectomy (MSV) has been proven to significantly improve semen quality, particularly microsurgical varicocelectomy, which is currently regarded as the preferred surgical approach.^6^ This technique enables precise identification and ligation of abnormal veins while preserving the testicular arteries and lymphatic systems, thereby minimizing recurrence rates and post‐operative complications.^7^ Studies have shown that this procedure can significantly improve semen parameters, such as sperm concentration, total sperm count, sperm motility, and sperm DNA fragmentation index, thus increasing the likelihood of natural conception.^8^, ^9^, ^10^, ^11^ According to a recent meta‐analysis by Fallara et al.,^12^ varicocelectomy significantly improved pregnancy rates (odds ratio [OR] = 1.29, 95% confidence interval [CI]: 1.00–1.65, *p* = 0.048) and sperm concentration (mean difference 12.34 million/mL, 95% CI 3.49–21.18, *p* = 0.006) compared to observation. Although no significant changes were noted in sperm motility and morphology overall, varicocelectomy markedly enhanced sperm concentration, motility, and morphology within the treatment group, especially in patients with abnormal semen parameters. On the other hand, intrauterine insemination (IUI) is commonly used to address mild male factor infertility in couples.^13^, ^14^ However, while IUI is less invasive and may improve pregnancy rates in the short term, it does not directly enhance semen quality. Therefore, for varicocoele patients with mild semen abnormalities, there remains clinical debate over whether surgery or IUI should be the preferred treatment option.

Currently, there is no universally accepted definition for “mild semen abnormalities,” and the criteria employed in various studies exhibit significant variation, creating challenges for clinical decision‐making. In the absence of an international consensus, we referred to multiple studies and defined a total motile sperm count (TMSC) of ≥5 million as the minimum threshold for natural conception or IUI.^15^, ^16^, ^17^, ^18^, ^19^, ^20^ In addition, based on the reference values provided in the *World Health Organization Laboratory Manual for the Examination and Processing of Human Semen* (5th edition),^21^ “mild semen abnormalities” were defined as cases where, under the condition of TMSC ≥ 5 million, at least one parameter—total sperm count, sperm concentration, or sperm motility—is below the World Health Organization (WHO) reference range. This definition offers a clearer diagnostic framework for future studies and provides stronger support for clinical decision‐making and efficacy evaluation.

Although varicocelectomy and IUI are widely used in the treatment of infertility, no studies have specifically compared the efficacy of these two approaches in varicocoele patients with mild semen abnormalities. To address this gap, we conducted a retrospective study aimed at evaluating clinical pregnancy and live birth rates following either MSV with natural conception or direct IUI treatment. Additionally, we assessed the impact of varicocelectomy on sperm concentration, total sperm count, and motility. By analyzing treatment outcomes in this specific population, our study aims to provide evidence‐based guidance on the optimal clinical management strategy—whether surgical intervention followed by natural pregnancy or immediate IUI—is more effective for varicocoele patients with mild semen abnormalities.

## 2 MATERIALS AND METHODS

### 2.1 Study population

**FIGURE 1:**
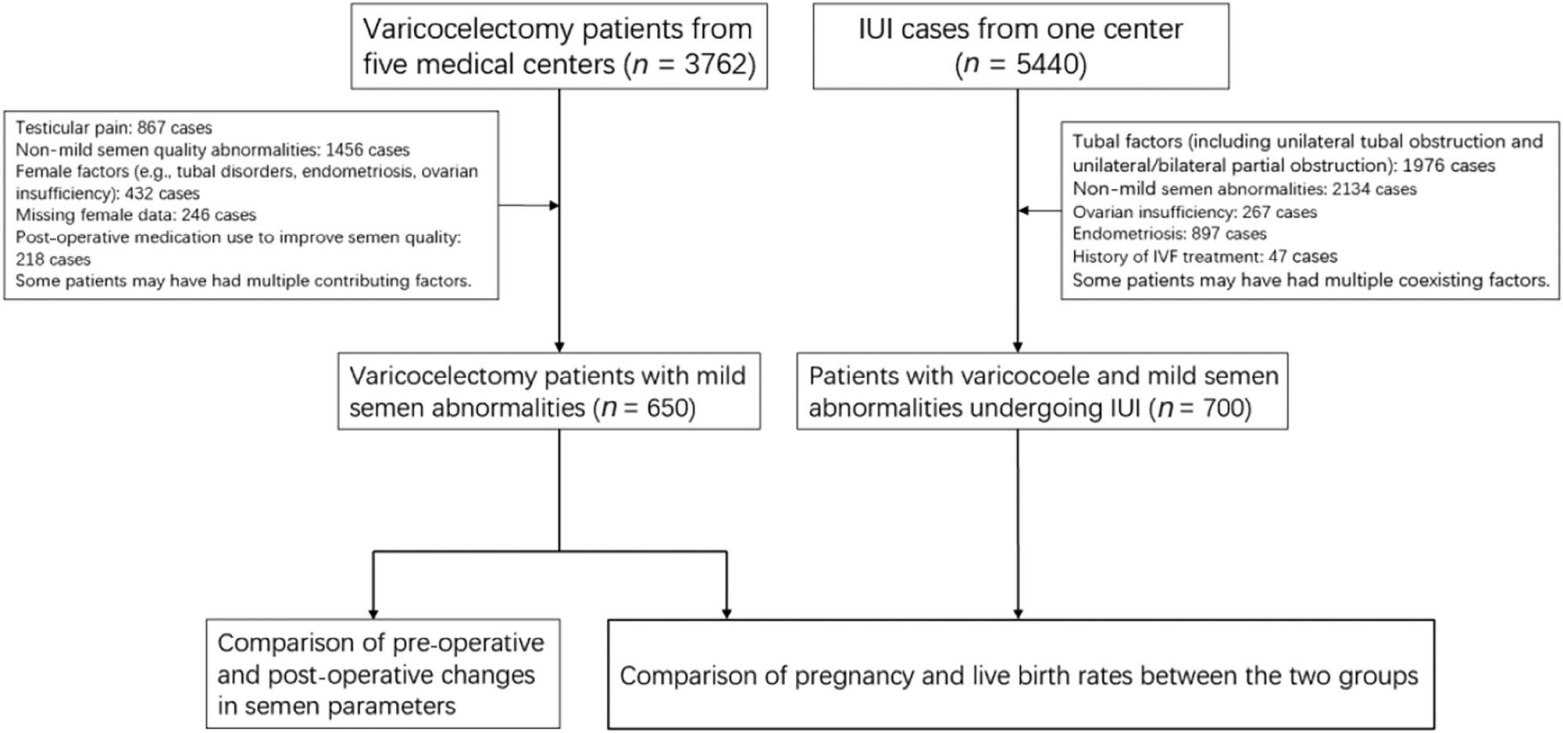
Flowchart of patient selection and exclusion for the varicocelectomy and intrauterine insemination (IUI) groups. IVF, in vitro fertilization.

This retrospective cohort study was conducted in accordance with the principles of the Helsinki Declaration and approved by the Institutional Review Board of the First Affiliated Hospital of Hainan Medical University (approval code: 2023‐KYL‐264, dated December 29, 2023). The study aimed to compare the efficacy of MSV and IUI in improving fertility in male patients with mild semen abnormalities and varicocoele, as well as to evaluate the impact of varicocelectomy on semen parameters. Data for the study were collected from multiple medical institutions. Varicocelectomy data were obtained from five centers: The First Affiliated Hospital of Hainan Medical University (*n* = 278), Sanya People's Hospital (*n* = 36), Danzhou Western Central Hospital (*n* = 58), Zunyi Huahuagang District People's Hospital (*n* = 68), and The First Affiliated Hospital of Nanhua University (*n* = 210). IUI data were sourced from The First Affiliated Hospital of Hainan Medical University (*n* = 700), which served as the single‐center provider of IUI data for this study. Data collection spanned from January 2015 to November 2023. The patient selection and exclusion process is illustrated in Figure 1.

### 2.2 Inclusion criteria

This study included male patients aged 22–45 years who were diagnosed with varicocoele by ultrasonographic evaluation of the male reproductive system (including scrotal ultrasound and transrectal ultrasonography) and andrologists and exhibited mild semen abnormalities based on semen analysis, following the WHO criteria. Specifically, patients with a TMSC > 5 × 10⁶ and at least one abnormal semen parameter were included. The definitions of semen abnormalities were based on the *WHO Laboratory Manual for the Examination and Processing of Human Semen* (5th edition)^21^: sperm concentration < 15 × 10⁶/mL, total sperm count < 39 × 10⁶ per ejaculate, or total motility < 40%. All patients had a history of infertility for at least 1 year. Their female partners were aged 20–38 years, with normal fertility function (normal ovarian reserve with anti‐Müllerian hormone [AMH] levels within the normal range) and confirmed tubal patency (assessed by hysterosalpingography).

### 2.3 Exclusion criteria

Patients with severe male infertility factors, such as azoospermia or total immotile spermatozoa, were excluded. Female partners with severe infertility issues, such as tubal obstruction, severe endometriosis, or ovarian failure, were also excluded. Additionally, patients who had undergone other assisted reproductive treatments (e.g., in vitro fertilization or intracytoplasmic sperm injection) within the past year were not eligible for the study. Patients with major systemic diseases or other conditions potentially affecting fertility, including infections of the reproductive tract (such as *Ureaplasma urealyticum*, *Chlamydia trachomatis*, and *Mycoplasma genitalium*), were excluded from the study.as well as those who used medications to improve semen quality after varicocelectomy, were also excluded.

### 2.4 IUI procedure

IUI was performed during either natural or stimulated cycles. Ovarian stimulation began on days 3–5 of the menstrual cycle, following standard protocols involving clomiphene citrate (CC), letrozole, or human menopausal gonadotropin (HMG)/human chorionic gonadotropin (HCG). For natural cycles or CC/letrozole‐stimulated cycles, follicular monitoring began on days 8–10 of the menstrual cycle. For HMG/HCG cycles, follicular monitoring started 3–5 days after initiating treatment. When follicles reached 18–20 mm in diameter, 5000–10,000 IU of HCG or a GnRH agonist was administered, followed by IUI 36 h later. Patients who did not experience menstruation within 14 days post‐IUI were instructed to undergo quantitative serum HCG testing. Positive HCG results were confirmed by transvaginal ultrasound 2 weeks later to assess fetal heartbeat and pregnancy location. The presence of a positive fetal heartbeat was considered a clinical pregnancy, which served as the primary outcome measure of this study.

### 2.5 Surgical procedure

MSV was performed under general anesthesia. Patients were placed in the supine position, and a 2–3 cm incision was made at the external inguinal ring. The skin and subcutaneous tissue were dissected, and Camper's and Scarpa's fasciae were incised to expose the spermatic cord below the external inguinal ring. The spermatic cord was elevated using Babcock forceps. Under 6–15× magnification, microsurgical procedures were performed. The external and internal spermatic fasciae were incised, and the vas deferens and accompanying vessels were isolated and protected with a plastic strip. Another rubber band was used to pull the isolated spermatic cord downward, with adjusted tension to ensure arterial blood flow, which was confirmed by observing vascular pulsation under the microscope. All identified arteries and lymphatics were preserved, while internal spermatic veins were doubly ligated and divided using 4‐0 silk sutures. The spermatic fasciae were closed with 4‐0 monofilament absorbable sutures, and the incision was closed in layers using the same sutures.

### 2.6 Data collection and follow‐up

The data for this study were collected from multiple medical institutions through electronic medical records and telephone follow‐ups, including baseline characteristics, diagnostic findings, and clinical outcomes. Baseline characteristics, such as patient age, body mass index (BMI), duration of infertility, AMH levels, and endocrine hormone levels, were obtained from electronic medical records. Semen parameters, including sperm concentration, TMSC, motility rate, and semen volume, were extracted from laboratory reports following the *WHO Laboratory Manual* (5th edition) to ensure accuracy and comparability, with sperm morphology parameters excluded to minimize variability. Infertility type (primary or secondary) and duration of infertility were determined based on patient history recorded in medical records, while varicocoele characteristics (laterality and grade) were obtained from physical examinations. In the varicocelectomy group, semen parameters were assessed at least three months post‐operatively to evaluate surgical effects, whereas in the IUI group, pregnancy and live birth outcomes were derived from up to three IUI treatment cycles within 1 year. Clinical pregnancy rate was defined as the proportion of patients who conceived at least once within the 1‐year follow‐up period, and live birth rate was defined as the proportion of those who achieved pregnancy within this period and were followed until delivery. Clinical pregnancy and live birth outcomes were collected through structured telephone follow‐ups and supplemented with electronic medical records when available, with pregnancy data further verified through telephone follow‐ups to ensure consistency. Additionally, a standardized approach was maintained for outcome definitions and follow‐up periods across both groups to enable valid comparisons.

### 2.7 Statistical analysis

Data analysis was performed using SPSS version 24.0. Continuous variables were presented as medians (interquartile ranges, IQR), and categorical variables as frequencies and percentages. Comparisons of continuous variables between groups were conducted using the Mann–Whitney *U* test based on data distribution. Comparisons of pregnancy and live birth rates between the two groups were conducted using the chi‐square test. Additionally, pregnancy and live birth rates were analyzed using univariate logistic regression to estimate ORs with 95% CIs. Variables significantly associated with outcomes in univariate analysis (*p* < 0.05) were included in multivariate logistic regression models to adjust for potential confounders. All statistical tests were two‐tailed, and *p* < 0.05 was considered statistically significant.

## 3 RESULTS

The baseline characteristics of 650 patients in the varicocelectomy group and 700 patients in the IUI group were compared. There were no significant differences between the two groups in terms of male age, BMI, duration of infertility, AMH levels, and baseline endocrine hormones (follicle‐stimulating hormone [FSH] and E2). Although the female partners in the IUI group were slightly older than those in the varicocelectomy group (*p* = 0.02), no significant differences were observed between the two groups in semen parameters (sperm concentration, sperm motility, and TMSC) (*p* > 0.05). Additionally, there were no significant differences in the laterality or grade distribution of varicocoele between the groups. Male BMI, smoking status, and alcohol consumption were also analyzed but showed no significant associations with clinical pregnancy or live birth outcomes. Overall, the baseline characteristics and semen quality of the two groups were comparable, providing a robust foundation for evaluating subsequent treatment outcomes (Table 1).

**TABLE 1:** Baseline characteristics of patients in the microsurgical varicocelectomy (MSV) and intrauterine insemination (IUI) groups.

Characteristic	MSV group (*n* = 650)	IUI group (*n* = 700)	*p*‐value
Male age, years (missing: 0, 0)	33 (28–37)	33 (30–36)	0.547
Female age, years (missing: 0, 0)	30 (27–34)	31 (28–33)	0.020
Duration of infertility, years (missing: 0, 0)	4 (2–5)	3 (2–5)	0.133
BMI (female), kg/m^2^ (missing:10, 2)	22.5 (18.7–26.4)	21.4 (19.6–23.7)	0.109
BMI (male), kg/m^2^ (missing: 0, 7)	25 (21.73–27.08)	24.2 (21.48–27.00)	0.369
Male smoking, % (missing: 0, 0)	43.8%	45.7%	0.526
Male drinking, % (missing: 0, 0)	18.4%	18.8%	0.907
Primary infertility, *n* (missing: 0, 0)	468	429	
Secondary infertility, *n* (missing: 0, 0)	182	271	
AMH (ng/mL) (missing: 8, 0)	4.67 (3.0–7.0)	4.56 (2.6–7.3)	0.658
FSH (female), IU/L (missing: 12, 0)	5.33 (3.39–7.38)	5.40 (4.28–6.63)	0.234
E2 (female), pg/mL (missing: 12, 0)	146 (107–199)	153 (95–214)	0.613
Laterality—left only (missing: 0, 0)	535	581	
Laterality—right only (missing: 0, 0)	0	1	
Laterality—bilateral (missing: 0, 0)	115	118	
Grade 1 (missing: 0, 0)	139	172	
Grade 2 (missing: 0, 0)	530	549	
Grade 3 (missing: 0, 0)	98	97	
Semen volume (mL) (missing: 0, 0)	2.6 (1.6–3.6)	2.8 (2.0–3.9)	< 0.05
Sperm concentration (million/mL) (missing: 0, 0)	35 (13–63)	30 (13–54)	0.1954
Total motility (PR + NP), % (missing: 0, 0)	26 (22–31)	26 (20–31)	0.09
Total motile sperm count (million) (missing: 0, 0)	18.6 (10.5–33.3)	19.3 (12.1–30.9)	0.545

*Note*: Values are presented as median (IQR) for continuous variables and frequency (%) for categorical variables. Statistical tests: Mann–Whitney *U* test for continuous variables, chi‐square test for categorical variables. Missing: (*x*, *y*) indicates *x* missing cases in the MSV group and *y* in the IUI group.

Abbreviations: AMH, anti‐Müllerian hormone; BMI, body mass index; E2, estradiol; FSH, follicle‐stimulating hormone; NP, non‐progressive motility; PR, progressive motility.

Among the 650 patients who underwent MSV, 41 did not provide post‐operative semen parameter data, leaving 609 patients for final analysis. The comparison of semen parameters showed that seminal volume remained unchanged before and after surgery (2.6 [1.6–3.6] mL vs. 2.4 [1.4–3.8] mL, *p* = 0.546). However, sperm concentration, total motility sperm ratio, and TMSC all showed significant improvements post‐operatively (Table 2).

**TABLE 2:** Pre‐ and post‐operative semen parameters in the MSV group.

Characteristic	Pre‐operation (*n* = 650)	Post‐operation (*n* = 609)	*p*
Semen volume (mL)	2.6 (1.6–3.6)	2.4 (1.4–3.8)	0.546
Sperm concentration (million /mL)	35.2 (13.2–63.2)	43.3 (25.4–70.9)	<0.001
Total motility (PR + NP) sperm ratio (%)	26 (22–31)	38 (32–44)	<0.001
Total motile sperm count (million)	18.84 (10.51–33.28)	34.91 (19.65–63.81)	<0.001

*Note*: Values are presented as median (IQR). Statistical test: Wilcoxon signed‐rank test for paired data. Missing data: 609 patients had both pre‐ and post‐operative semen samples; 41 were excluded because of incomplete data.

Abbreviations: MSV, microsurgical subinguinal varicocelectomy; NP, non‐progressive motility; PR, progressive motility.

In the varicocelectomy group, 229 patients achieved pregnancy, yielding a pregnancy rate of 35.23% (229/650), and 202 patients achieved live births, resulting in a live birth rate of 31.08% (202/650). In the IUI group, 207 patients achieved pregnancy, corresponding to a pregnancy rate of 29.57% (207/700), and 168 patients had live births, resulting in a live birth rate of 24.00% (168/700) (Table 3).

**TABLE 3:** Pregnancy and live birth rates in the IUI and MSV groups.

Group	IUI group (*n* = 700)	MSV group (*n* = 650)	*p*
Pregnancy rate (%)	29.57% (207/700)	35.23 (229/650)	0.0305
Live birth rate (%)	24.00% (168/700)	31.08 (202/650)	0.0043

*Note*: Values are presented as *n* (%). All patients completed follow‐up. Statistical test: Chi‐square test. Missing data: None.

Abbreviations: IUI, intrauterine insemination; MSV, microsurgical subinguinal varicocelectomy.

In the univariate logistic regression analysis of pregnancy rates, with the IUI group as the reference group, varicocelectomy significantly increased the odds of clinical pregnancy (OR = 1.30, 95% CI: 1.04–1.64, *p* = 0.026) and live birth (OR = 1.72, 95% CI: 1.01–2.94, *p* = 0.042). Additionally, increasing male age (OR = 0.97 per year increase, 95% CI: 0.95–0.99, *p* = 0.005) and female age (OR = 0.95 per year increase, 95% CI: 0.92–0.98, *p* = 0.0006) were negatively associated with pregnancy outcomes, indicating that older age is associated with decreased reproductive success., seminal volume (OR = 1.10, 95% CI: 1.02–1.18, *p* = 0.016), infertility type (primary vs. secondary, OR = 1.79, 95% CI: 1.41–2.27, *p* < 0.001), and duration of infertility (OR = 0.81, 95% CI: 0.76–0.85, *p* < 0.001) were also significantly associated. In the multivariate logistic regression analysis, treatment group (OR = 0.70, 95% CI: 0.55–0.89, *p* = 0.004), female age (OR = 0.97, 95% CI: 0.94–1.00, *p* = 0.043), infertility type (OR = 1.99, 95% CI: 1.55–2.55, *p* < 0.001), and duration of infertility (OR = 0.81, 95% CI: 0.77–0.86, *p* < 0.001) remained statistically significant (Table 4).

**TABLE 4:** Univariable and multivariable analysis of factors associated with pregnancy and live birth rates.

	UVA (pregnancy rate)		MVA (pregnancy rate)		UVA (live birth)		MVA (live birth)	
Variable	OR (95% CI)	*p*‐value	OR (95% CI)	*p*‐value	OR (95% CI)	*p*‐value	OR (95% CI)	*p*‐value
Treatment group (IUI = 1, MSV = 0)	0.77 (0.61, 0.97)	0.026	0.70 (0.55, 0.89)	0.004	0.58 (0.34, 0.98)	0.042	0.53 (0.30, 0.94)	0.029
Male age	0.97 (0.95, 0.99)	0.005	0.99 (0.96, 1.01)	0.286	0.96 (0.91, 1.00)	0.073		
Female age	0.95 (0.92, 0.98)	0.0006	0.97 (0.94, 1.00)	0.043	0.88 (0.83, 0.94)	<0.001	0.88 (0.82, 0.94)	<0.001
Female BMI	1.00 (0.98, 1.04)	0.755			0.97 (0.93, 1.02)	0.092		
Male BMI	1.00 (0.97, 1.03)	0.897			1.01 (0.93, 1.08)	0.884		
Male smoking	1.15 (0.90, 1.46)	0.260			1.01 (0.60, 1.71)	0.961		
Male drinking	1.10 (0.81, 1.49)	0.538			1.38 (0.67, 2.83)	0.384		
AMH	0.99 (0.96, 1.03)	0.701			1.12 (1.01, 1.23)	0.028	1.10 (0.99, 1.22)	0.067
FSH	1.02 (0.97, 1.07)	0.461			0.97 (0.87, 1.09)	0.642		
E2	1.00 (1.00, 1.00)	0.485			1.00 (0.997, 1.004)	0.892		
Semen volume	1.10 (1.02, 1.18)	0.016	1.07 (0.99, 1.16)	0.079	1.13 (0.95, 1.34)	0.171		
Sperm concentration	1.00 (1.00, 1.00)	0.708			1.01 (0.995, 1.015)	0.309		
PR + NP	1.00 (0.99, 1.01)	0.901			0.997 (0.977, 1.018)	0.787		
PR + NP sperm number	1.00 (1.00, 1.01)	0.311			1.01 (0.996, 1.027)	0.161		
Infertility status	1.79 (1.41, 2.27)	<0.001	1.99 (1.55, 2.55)	<0.001	0.87 (0.52, 1.48)	0.619		
Duration of infertility	0.81 (0.76, 0.85)	<0.001	0.81 (0.77, 0.86)	<0.001	1.03 (0.90, 1.19)	0.640		
Unilateral/bilateral (Uni = 1, Bi = 0)	1.13 (0.84, 1.54)	0.419			0.69 (0.31, 1.51)	0.347		
Grade 1	0.87(0.65, 1.16)	0.355			0.995 (0.505, 1.962)	0.989		
Grade 2	1.01 (0.79, 1.30)	0.913			0.71 (0.39, 1.29)	0.265		
Grade 3	1.19 (0.85, 1.63)	0.311			2.08 (0.80, 5.42)	0.132		

*Note*: Values are odds ratios (OR) with 95% confidence intervals (CI). Multivariable models include variables with *p* < 0.05 in univariable analysis. Reference group for treatment: IUI. Missing data: Participants with incomplete covariate data were excluded from multivariable analysis. Statistical method: Logistic regression.

Abbreviations: AMH, anti‐Müllerian hormone; BMI, body mass index; E2, estradiol; FSH, follicle‐stimulating hormone; IUI, intrauterine insemination; MSV, microsurgical subinguinal varicocelectomy; MVA, multivariable analysis; NP, non‐progressive motility; PR, progressive motility; UVA, univariable analysis.

For live birth rates, univariate logistic regression analysis showed that treatment group (OR = 0.58, 95% CI: 0.34–0.98, *p* = 0.042), female age (OR = 0.88, 95% CI: 0.83–0.94, *p* < 0.001), and AMH levels (OR = 1.12, 95% CI: 1.01–1.23, *p* = 0.028) were significantly associated with live birth outcomes. In the multivariate logistic regression analysis, treatment group (OR = 0.53, 95% CI: 0.30–0.94, *p* = 0.029) and female age (OR = 0.88, 95% CI: 0.82–0.94, *p* < 0.001) remained statistically significant, while AMH levels (OR = 1.10, 95% CI: 0.99–1.22, *p* = 0.067) showed a trend toward significance (Table 4).

## 4 DISCUSSION

This study aimed to investigate the differences in efficacy between MSV and IUI in patients with mild semen abnormalities and varicocoele. The results demonstrated that clinical pregnancy rates and live birth rates were significantly higher in the MSV group compared to the IUI group. Furthermore, MSV showed significant improvement in sperm concentration, TMSC, and sperm motility. Female age, infertility type, and duration of infertility were also confirmed to have significant impacts on reproductive outcomes.

All varicocoele patients in this study presented with mild semen abnormalities, a group for which there is ongoing clinical debate regarding whether IUI or surgical intervention should be the first‐line treatment. IUI is favored for its simplicity and shorter treatment cycle, and some couples with mild male factor infertility can achieve pregnancy within a relatively short period. However, IUI does not fundamentally improve semen parameters, and multiple IUI failures often necessitate transitioning to in vitro fertilization or intracytoplasmic sperm injection. In contrast, MSV, despite involving surgical risks, allows precise identification and ligation of abnormal veins under microscopic guidance, effectively preserving the testicular arteries and lymphatic vessels. This approach minimizes recurrence and complications while significantly improving semen quality.^3^ Our findings indicate that for patients with mild semen abnormalities and varicocoele, the MSV group had superior pregnancy and live birth rates compared to the IUI group, consistent with other studies confirming the role of MSV in improving sperm quality and natural pregnancy rates.^8^, ^10^, ^22^, ^23^

Our multivariate analysis highlighted that, compared with IUI (reference group), varicocelectomy independently improved clinical pregnancy (OR = 1.43, 95% CI: 1.12–1.83, *p* = 0.004) and live birth rates (OR = 1.56, 95% CI: 1.21–2.02, *p* < 0.05). These results strongly support the clinical advantage of varicocelectomy over IUI in improving reproductive outcomes for patients with mild semen abnormalities. It is noteworthy that multivariate analysis revealed that female age had a significant impact on pregnancy and live birth rates, aligning with previous reports that increasing female age is associated with reduced IUI success rates.^24^, ^25^, ^26^, ^27^ In this study, the female partners in the IUI group were slightly older than those in the MSV group, which may have somewhat compromised the outcomes of the IUI group. However, even after adjusting for female age and other potential confounding factors, the MSV group still demonstrated significantly higher pregnancy and live birth rates, suggesting that varicocelectomy contributes independently to improving overall reproductive outcomes.

The study also found that infertility type (primary vs. secondary) and duration of infertility were significantly associated with IUI pregnancy rates. Patients with secondary infertility, having had prior successful pregnancies or live births, may possess inherent reproductive potential in terms of male sperm function and/or female fertility conditions, resulting in higher pregnancy probabilities. Similar observations have been reported in other infertility studies.^28^, ^29^ Moreover, multiple studies have shown that longer durations of infertility are associated with lower IUI pregnancy rates,^30^, ^31^ a finding corroborated by our study. These results underscore the importance of considering infertility type and duration when managing infertility, to better predict prognosis and develop personalized treatment plans.

It should also be noted that among male‐related predictors, primary infertility was associated with poorer outcomes compared to secondary infertility. However, not all factors that may affect fertility in men with primary infertility were included in our dataset, such as serum inhibin B, testicular volume, and FSH/LH ratio. Since our study population primarily consisted of men with mild semen abnormalities, routine hormonal or morphological assessments are not always clinically indicated. Nevertheless, we acknowledge this as a limitation, and future prospective studies should consider more detailed characterization of primary infertility to identify underlying causes and optimize treatment strategies.

Regarding improvements in semen parameters, aside from seminal volume, the MSV group exhibited significant enhancements in sperm concentration, progressive motility, and TMSC. These findings align with previous studies confirming the positive effects of MSV on semen quality.^3^, ^7^, ^17^

This study, while retrospective in design, has notable strengths, including a large sample size and data sourced from multiple medical institutions. However, several limitations should be noted. First, as a retrospective study, some potential confounding factors may not have been fully controlled, such as variations in diagnostic and surgical techniques among institutions. Second, differences in follow‐up duration and adherence among patients may have influenced the final statistical analysis. Third, there is no globally accepted definition of “mild semen abnormalities” in clinical practice. Based on multiple literature reports and the WHO 5th edition standards, we defined mild abnormalities as cases with TMSC ≥ 5 × 10⁶ and at least one parameter (total sperm count, sperm concentration, or motility) below the WHO reference ranges. Although this definition has clinical relevance, the lack of global consensus necessitates further validation through prospective, multicenter, and large‐scale studies.

In conclusion, this study demonstrates that for male patients with mild semen abnormalities and varicocoele, MSV is more effective than IUI in improving pregnancy and live birth rates, while also enhancing semen parameters. We recommend that clinicians consider female partner age, infertility type, and duration of infertility when treating such patients. Future large‐scale, multicenter, prospective randomized controlled trials are needed to further validate the long‐term safety and efficacy of different treatment strategies, thereby developing more targeted, individualized management plans for patients with mild semen abnormalities and varicocoele.

## AUTHOR CONTRIBUTIONS

**Yanlin Ma**: Supervision; writing—review and editing; funding acquisition. **Weiming Deng**: Resources; investigation; project administration; data interpretation; manuscript revision. **Tingting Wu**: Validation; investigation; formal analysis; writing—original draft. **Qi Li**: Funding acquisition; supervision; formal analysis. **Yuanjie Li**: Resources; data curation. **Wenjie Mai**: Resources; writing—review and editing. **Lizheng Yan**: Resources; investigation. **Changwei Liang**: Resources. **Lingxiao Zhang**: Conceptualization; methodology; supervision; writing—original draft; writing—review and editing.

## CONFLICT OF INTEREST STATEMENT

The authors declare no conflicts of interest.
